# Artificial intelligence analysis applied to the treatment of granulosa cell tumors of the ovary

**DOI:** 10.3389/frai.2025.1675969

**Published:** 2025-11-11

**Authors:** OPhir Nave, Pnina Barasheshet

**Affiliations:** 1 Department of Mathematics, Jerusalem College of Technology (Academic Lev Center), Jerusalem, Israel; 2 Department of Computer Science, The College of Management Academic Studies, Rishon LeZion, Israel; 3 Faculty of Medicine, Ben-Gurion University of the Negev, Be'er Sheva, Israel

**Keywords:** artificial intelligence, mathematical model, PAC-1, oncolytic virus, granulosa cells, ovarian cancer, machine learning

## Abstract

**Introduction:**

Granulosa cell tumors (GCTs) of the ovary are rare malignancies with limited systemic treatment options and high recurrence rates. Combining tumor necrosis factor-related apoptosis-inducing ligand (TRAIL)-producing oncolytic viruses with procaspase-3 activator (PAC-1) presents a promising therapeutic strategy, as TRAIL initiates apoptosis while PAC-1 amplifies caspase activity. However, patient responses remain variable, necessitating predictive frameworks that can integrate biological complexity with clinical data.

**Methods:**

We developed a hybrid framework that integrates a mechanistic mathematical model of TRAIL-oncolytic virus and PAC-1 therapy with machine learning (ML) algorithms to predict tumor dynamics in GCTs. Four datasets (continuous and categorical tumor size measurements) were analyzed. Clinical and imaging data were merged with individualized solutions from the mathematical model to generate enriched feature sets for ML training. Linear regression and neural network models were trained and evaluated using accuracy, F1 scores, and root mean square error (RMSE).

**Results:**

Integrating mathematical model outputs improved predictive performance across all datasets. Linear regression models showed reduced RMSE compared to models without mathematical features (e.g., RMSE decreased from 18.4 to 16.1 in one dataset). Neural networks incorporating model-derived variables achieved higher accuracy and F1 scores (e.g., accuracy improved from 77.3% to 91.4%). Sensitivity analysis revealed that tumor proliferation and apoptosis rates were the most influential parameters for treatment outcomes.

**Discussion:**

Our results demonstrate that coupling mathematical modeling with ML enhances the prediction of tumor burden in patients undergoing TRAIL-oncolytic virus and PAC-1 therapy. This integrative approach provides mechanistic insight into tumor behavior while improving predictive accuracy, supporting the development of personalized therapeutic strategies for GCTs. The framework also offers broader applicability to other cancers with limited treatment options and heterogeneous responses.

## 1 Introduction

Granulosa cell tumors (GCTs) of the ovary constitute a rare subtype of ovarian neoplasms, accounting for approximately 2%–5% of all ovarian malignancies (Colombo et al., 2012). These tumors arise from sex cord-stromal tissue and are notable for their distinct biological behavior: they generally grow slowly yet retain a striking propensity for very late recurrence, even decades after apparently successful primary treatment (Van Meurs et al., 2014, 2013). For patients with early-stage disease, surgical resection remains the cornerstone of management. However, once recurrence or advanced disease develops, the clinical scenario becomes considerably more challenging. Unlike epithelial ovarian cancers, for which multiple systemic regimens are available, recurrent GCTs lack effective systemic treatment options. Platinum-based chemotherapy, often adapted from epithelial ovarian cancer protocols, has shown only limited and transient benefit (Van Meurs et al., 2013; Homesley et al., 1999; Bhat et al., 2024), while hormonal and radiotherapy approaches provide inconsistent responses (Van Meurs et al., 2014). Consequently, many patients endure repeated surgeries with significant morbidity, and no curative systemic therapy exists. This therapeutic gap highlights a pressing unmet clinical need: there are currently no approved targeted or precision therapies that reliably improve outcomes in GCTs.

GCTs represent a particularly compelling tumor type in which to establish a proof-of-concept for novel therapeutic frameworks. First, their biology is characterized by apoptotic dysregulation, with elevated procaspase-3 levels and a relative susceptibility to extrinsic apoptotic signaling, making them uniquely suited for apoptosis-inducing strategies such as TRAIL-producing oncolytic viruses and PAC-1 (Russell et al., 2012; Ashkenazi, 2008; Aziz et al., 2010). Second, compared with highly heterogeneous epithelial ovarian cancers, GCTs display a more uniform molecular landscape, providing a tractable model system for developing integrative predictive approaches. Third, the rarity of GCTs creates both a challenge and an opportunity: conventional large-scale clinical trials are difficult to conduct, increasing the value of computational models that can extract maximal insight from limited clinical datasets. Finally, because GCTs exemplify tumors with indolent growth but unpredictable recurrence and resistance to standard therapies, they offer a clinically meaningful setting to test strategies that combine mechanistic modeling with machine learning to personalize therapy.

In recent years, targeted combination therapies have emerged as promising strategies for GCTs and other refractory cancers. Tumor necrosis factor-related apoptosis-inducing ligand (TRAIL)-producing oncolytic viruses selectively replicate in tumor cells while sparing normal tissue, delivering TRAIL to the tumor microenvironment and activating extrinsic apoptotic pathways (Russell et al., 2012). Procaspase-3 activator 1 (PAC-1) directly activates procaspase-3, a key executioner of apoptosis, and synergizes with pro-apoptotic agents, such as TRAIL, to amplify tumor cell death (Ashkenazi, 2008; Aziz et al., 2010; Reed, 1999; Peterson et al., 2009). The rationale for combining TRAIL-oncolytic viruses with PAC-1 is therefore compelling: TRAIL initiates apoptosis upstream, while PAC-1 amplifies downstream caspase activity, together providing a potent and tumor-selective pro-apoptotic strategy (Wang and El-Deiry, 2003; Gujar et al., 2018).

Despite this strong biological rationale, patient responses to such combination therapies remain highly variable, reflecting tumor heterogeneity, viral dynamics, drug pharmacokinetics, and host immune responses (Lin et al., 2023; Esteva et al., 2019). Accurate prediction of therapeutic outcomes in GCTs thus requires new frameworks that can integrate complex, multidimensional data. Artificial intelligence (AI), particularly machine learning (ML), offers a means to identify hidden patterns in clinical, molecular, and imaging data that are not discernible through traditional methods (Rockne et al., 2008; Zhang et al., 2017). Integrating mechanistic, mathematical models of tumor growth, viral kinetics, and drug action into ML pipelines enables the development of hybrid, predictive models that not only forecast treatment outcomes, but also provide mechanistic insights (Obermeyer and Emanuel, 2016; Le Sauteur-Robitaille et al., 2023).

In this study, we present an artificial analysis framework that combines a mathematical model of TRAIL-oncolytic virus and PAC-1 therapy with ML algorithms to predict tumor dynamics in ovarian GCTs. By training ML models on clinical and imaging data enriched with personalized mathematical model outputs, we aim to improve predictive accuracy and support the design of more effective, individualized treatment strategies. GCTs, with their well-defined unmet need and distinctive biology, provide an ideal proof-of-concept setting for this integrated modeling approach, with potential relevance to other difficult-to-treat malignancies.

## 2 Mathematical model

In this section, we present the mathematical model describing granulosa cell tumors of the ovary treatment by a combination of a TRAIL-producing oncolytic virus and PAC-1. The mathematical model includes nonlinear ordinary differential equation of the first order. The assumptions of the model are as follows (Le Sauteur-Robitaille et al., 2023):

GCT Equations 1–5: The variables in the granulosa cell tumor (GCT) model are defined as follows: Q, the number of quiescent tumor cells; *G*_1_, the number of cells in the *G*_1_ phase; and *A*_*i*_ (*i* = 1…, *n*), the *i*^th^ compartment of the active phases of the cell cycle, with *N* denoting the total number of active compartments. Quiescent cells transition into the *G*_1_ phase at a rate of *a*_1_, progress into the active phases at *a*_2_, and undergo apoptosis at *d*_2_. Upon entering the first active compartment, *A*_1_, at rate *a*_2_, cells sequentially transit through additional active compartments, *A*_*i*_, at rate *k*_*tr*_. Throughout these active compartments, cells may also undergo apoptosis at a rate of *d*_3_.

OV Equations 6–7: The variables of the oncolytic virus (OV) are denoted by *I*, the infected cells, and *V*, the viral particles. The infected cells are generated through mass-action contact dynamics between viral particles and cells in the *G*_1_ phase, and active phases of the cell cycle *N*. This interaction occurs at a rate of κ*ηV*, which accounts for the half-maximal effective concentration of virions, η_0.5_.

Tumor-innate immune interactions Equations 8–9: The variables that describe the interaction between the tumor-innate and immune are Cytokine, *C*, and the population of phagocytes, *P*. The set of equations of these variables are incorporate parameters such as the rate with tumor cells, *k*_*p*_, and the digestion rates of these immune cells, *k*_*Q*_ and *k*_*s*_. Additionally, immune activation was incorporated by modeling the recruitment and stimulation of phagocytes at the site of oncolytic virus infection, driven by cytokine signaling. Cytokines are produced at a rate of *C*_*prod*_ in response to the number of infected cells *I* and are eliminated at a rate of *k*_*elim*_. The cytokine-phagocyte interaction modulates the population of tumor-targeting phagocytes at a rate of $\phi \left(C\right)=\frac{k_{cp}C}{C_{0.5}+C}$, while these immune cells undergo natural cell death at a rate of γ_*P*_.

Pharmacokinetics of PAC-1 Equations 10–12: The variables that describe the treatment of a combination of PAC-1 and TRAIL are *P*_*A*_, *P*_*PAC*−1_, and *P*_*e*_. The administration process of PAC-1 was modeled with the dose initially entering the gastrointestinal tract, *P*_*A*_ before being absorbed into the bloodstream, *P*_*PAC*−1_ at a rate of *k*_*a*_. After entering the plasma, PAC-1 is cleared at a rate of *k*_*ep*_ and distributed to the peripheral compartment, *P*_*e*_, with the exchange governed by the transit parameters *k*_12*P*_ and *k*_21*P*_.

Pharmacokinetics of TRAIL Equations 13–15: The variables that describe the TRAIL administration are *T*, *T*_*P*_, and *T*_*A*_. The pharmacokinetics (PK) of TRAIL were described using an irreversible binding, target-mediated drug disposition (TMDD) model, assuming a constant receptor count, *R*_0_. This model incorporates three compartments: the free TRAIL ligand, *T*, the receptor-bound TRAIL complex, *T*_*P*_, and the ligand present in the peripheral tissues, *T*_*A*_. TRAIL is generated at a rate of α_*T*_ from the lysis of infected cells, and continuously at a constant rate, *T*_*prod*_. Its elimination occurs at a rate of *k*_*el*_. TRAIL binds to death receptors, forming a complex at a rate of *k*_*on*_, and it moves between the ligand compartment *T*_*A*_ with transition rates *k*_12_ and *k*_21_. Once the complex is formed, it undergoes degradation at a rate of *k*_*int*_.

Based on the above assumptions, the mathematical model includes the following ODE system of equations. All dynamical variables, parameters, and their corresponding units are provided in Tables 1–3.


$$\begin{array}{l} \frac{dQ}{dt}=2k_{tr}A_{j}-a_{1}Q-\frac{k_{p}P}{1+k_{Q}Q}Q, \end{array}$$ (1)

$$\begin{array}{l} \frac{dG_{1}}{dt}=a_{1}Q-\left(a_{2}+d_{2}\left(1+E\right)+\kappa \eta V+\frac{k_{p}P}{1+k_{s}G_{1}}\right)G_{1}, \end{array}$$ (2)

$$\begin{array}{l} \frac{dA_{1}}{dt}=a_{1}G_{1}-k_{tr}A_{1}-\left(d_{3}\left(1+E\right)+\kappa \eta V+\frac{k_{p}P}{1+k_{s}G_{1}}\right)A_{1}, \end{array}$$ (3)

$$\begin{array}{l} \frac{dA_{i}}{dt}=k_{tr}\left(A_{i-1}-A_{i}\right)-\left(d_{3}\left(1+E\right)+\kappa \eta V+\frac{k_{p}P}{1+k_{s}G_{1}}\right)A_{i}, \end{array}$$ (4)

$$\begin{array}{l} \frac{dN}{dt}=a_{2}G_{1}-\frac{k_{tr}}{a_{2}}A_{n}-\left(d_{3}\left(1+E\right)+\kappa \eta V+\frac{k_{p}P}{1+k_{s}G_{1}}\right)N, \end{array}$$ (5)

$$\begin{array}{l} \frac{dI}{dt}=\kappa \eta V\left(G_{1}-N\right)-\delta I, \end{array}$$ (6)

$$\begin{array}{l} \frac{dV}{dt}=\alpha \delta I-\omega V-\kappa \eta V\left(G_{1}-N\right), \end{array}$$ (7)

$$\begin{array}{l} \frac{dC}{dt}=C_{prod}-k_{elim}C, \end{array}$$ (8)

$$\begin{array}{l} \frac{dP}{dt}=\frac{k_{cp}C}{C_{0.5}+C}-\gamma_{p}P, \end{array}$$ (9)

$$\begin{array}{l} \frac{dP_{A}}{dt}=-k_{a}P_{A}, \end{array}$$ (10)

$$\begin{array}{l} \frac{dP_{PAC-1}}{dt}=\frac{k_{a}P}{V_{PAC-1}}-\left(k_{ep}-k_{12P}\right)P_{PAC-1}+k_{21P}P_{e}, \end{array}$$ (11)

$$\begin{array}{l} \frac{dP_{e}}{dt}=k_{12P}P_{PAC-1}-k_{21P}P_{e}, \end{array}$$ (12)

$$\begin{array}{l} \frac{dT}{dt}=\alpha_{T}\delta I-k_{el}T-k_{on}T\left(R_{0}-T_{P}\right)-k_{12}T+k_{21}\frac{T_{A}}{V_{T}}\\ +T_{prod}, \end{array}$$ (13)

$$\begin{array}{l} \frac{dT_{P}}{dt}=k_{on}R_{0}T-\left(k_{int}-k_{on}T\right)T_{P}, \end{array}$$ (14)

$$\begin{array}{l} \frac{dT_{A}}{dt}=k_{12}TV_{T}-k_{21}T_{A}. \end{array}$$ (15)

The initial conditions of the mathematical model are


$$\begin{array}{l} Q\left(0\right)=Q_{0},G_{1}\left(0\right)=G_{1,0},A_{1}\left(0\right)=A_{1,0},A_{i}\left(0\right)=A_{i,0},\\ N\left(0\right)=N_{0},I\left(0\right)=I_{0}V\left(0\right)=V_{0},C\left(0\right)=C_{0},P\left(0\right)=P_{0},\\ P_{A}\left(0\right)=P_{A,0},P_{PAC-1}\left(0\right)=P_{PAC-1,0},P_{e}\left(0\right)=P_{e,0},\\ T\left(0\right)=T_{0}T_{P}\left(0\right)=T_{P,0},T_{A}\left(0\right)=T_{A,0}. \end{array}$$ (16)

**Table 1:** List of parameters for the model.

**Parameters**	**Units**	**Descriptions**	**Values**	**Sources**
*a*_1_	1/day	*Q* to *G*_1_ rate	3.3498	Fit from data
*a*_2_	1/day	*G*_1_ to *A*_1_ rate	1.44	Fit from data
*d*_2_	1/day	*G*_1_ apoptotic rate	0.2	Fit from data
*d*_3_	1/day	Active phase apoptotic rate	0.1732	Calculated
*k*_*tr*_	1/day	Active phase transfer rate	8.4540	Calculated
κ	1/day	Virion infection rate	0.054	Jenner et al., 2021
δ	1/day	Lysis rate	2.48	Jenner et al., 2021
α	Virions/cell	Burst size	1.12	Jenner et al., 2021
ω	1/day	Virion decay rate	40.3	Jenner et al., 2021
*k*_*p*_	1/day	Phagocyte-tumor cell contact rate	9.23	Jenner et al., 2021
*k*_*q*_, *k*_*s*_	–	Phagocyte cell digestion constant	0.064	Jenner et al., 2021
Ψ_1/2_	10^10^cells/day	Cytokine production half-effect	0.00011	Jenner et al., 2021
*k*_*cp*_	10^10^cells/day	Maximal immune cell production rate	4.6754	Jenner et al., 2021
η_1/2_	Virions	Virion half-effect concentration	0.51	Jenner et al., 2021
*C*_1/2_	ng/ml/day	Phagocyte production half-effect	0.739	Jenner et al., 2021
γ_*P*_	1/day	Phagocyte death rate	0.35	Jenner et al., 2021
$C_{prod}^{*}$	ng/ml/day	Homeostatic cytokine production rate	3.9863 × 10^−4^	Jenner et al., 2021
$C_{prod}^{\max}$	ng/ml/day	Maximal cytokine production rate	1.429	Jenner et al., 2021
*k*_*elim*_	1/day	Cytokine elimination rate	0.16139	Jenner et al., 2021
*j*	–	Number of transit compartments	6	Calculated
τ	Days	Expected cell cycle duration	0.7097	Calculated
*T* ^*^	ng/ml	Homeostatic TRAIL concentration	0.08090	Xiang et al., 2014

Contains cell growth parameters, viral parameters and immune system parameters, along with other necessary values.

**Table 2:** List of PK parameters.

**Parameters**	**Units**	**Descriptions**	**Values**	**Sources**
*k*_*a*_	1/day	PAC-1 oral absorption rate	2.96	Fit using data from Danciu et al., 2023
*V*_*PAC*_	ml	Volume of PAC-1 compartment	3390.45	Fit using data from Danciu et al., 2023
*k*_*ep*_	1/day	PAC-1 elimination rate	61.97	Fit using data from Danciu et al., 2023
*k*_12*P*_	1/day	Transfer rate from *PAC* to *P*_*e*_	183.49	Fit using data from Danciu et al., 2023
*k*_21*P*_	1/day	Transfer rate from *P*_*e*_ to *PAC*	1.18	Fit using data from Danciu et al., 2023
α_*T*_	ng/ml/cell	TRAIL production from virus	7.5837 × 10^−6^	Fit using data from Oh et al., 2018
*k*_*el*_	1/day	TRAIL elimination rate	45	Fit using data from Kelley et al., 2001
*k*_*on*_	1/day	TRAIL binding rate	0.026	Fit using data from Kelley et al., 2001
*R*_0_	ng/ml	Initial bound TRAIL and receptor complex target concentration	457.49	Fit using data from Kelley et al., 2001
*k*_12_	1/day	Transfer rate from *T* to *T*_*A*_	11.38	Fit using data from Kelley et al., 2001
*k*_21_	1/day	Transfer rate from *T*_*A*_ to *T*	0.0043	Fit using data from Kelley et al., 2001
*V*	ml	Volume of TRAIL main compartment	100.04	Fit using data from Kelley et al., 2001
*k*_*int*_	1/day	Bound TRAIL Internalization rate	22.15	Fit using data from Kelley et al., 2001

Contains parameters for the PAC-1 two-compartment model and the TRAIL TMDD model.

**Table 3:** List of PD parameters.

**Parameters**	**Units**	**Description**	**Values**	**Sources**
*E*_*max, PAC*_	–	Maximum efficacy of PAC-1	0.8764	Cardinal et al., 2022
*E*_*max, TRAIL*_	–	Maximum efficacy of TRAIL	0.438	Cardinal et al., 2022
*EC*50_*PAC*_	ng/ml	PAC-1 half-effect concentration	1,176.7	Calculated from Cardinal et al., 2022
*EC*50_*TRAIL*_	ng/ml	TRAIL half-effect concentration	5	Cardinal et al., 2022
γ_*PAC*_	–	PAC-1 hill coefficient	1.35	Cardinal et al., 2022
γ_*TRAIL*_	–	TRAIL hill coefficient	0.874	Cardinal et al., 2022
Ψ	–	Potency	0.8	Fit using data from Cardinal et al., 2022

Parameters necessary to the joint effect function of PAC-1 plus TRAIL- producing OV.

## 3 The dataset

### 3.1 Datasets with tumor size as a continuous variable

In this study, which focuses on the treatment of granulosa cell tumors of the ovary through the combined action of a TRAIL-producing oncolytic virus and PAC-1 therapy, we employed machine learning (ML) algorithms to enhance the prediction of tumor dynamics. Four datasets were analyzed in conjunction with mathematical models to improve the accuracy of tumor size prediction. Two of these datasets contained tumor size as a continuous variable, while the other two reported tumor size categorically (divided into tertiles).

The first dataset involved 10, 389 women receiving neoadjuvant chemotherapy for ovarian cancer, with detailed clinical and demographic data, including ethnicity, ovarian laterality, age at *MRI*1 (in years), subtype (lymph node-positive, *PIK*3*CA* mutation, *BRCA* mutation, and *TP*53 mutation), and *BMI*. Tumor sizes were recorded by MRI at 4 time points and measured by the longest diameter (*LD* in *cm*) and volume 4 (*cc*).

The second dataset consisted of 25, 985 women diagnosed with stage 2 or 3 ovarian cancer, recording tumor size at 3 MRI time points, along with clinical information.

The primary objective was to predict tumor size at each time point as accurately as possible, supporting the optimization of a TRAIL-producing oncolytic virus and PAC-1 therapy. To achieve this, we incorporated immunological features known to influence tumor behavior, such as *CD*4 + *T* cells, *T* − *reg* cells (*Dentritic* cells), and treatment parameters. Due to challenges in direct patient measurement, these features were derived from a mathematical model.

This model describes immune responses to chemotherapy (*AC*), refined for dosage and timing precision. The data were then pruned to include only treatment-matched samples, resulting in refined datasets of 10, 389 and 25, 389 samples, respectively.

The clinical data were merged with the mathematical model outputs using the initial MRI tumor size as *T*_0_. Individualized solutions were computed using the *ODE*45 Matlab function, producing unique solution vectors for each woman at 3 time points for variables such as *N* (*NK* cells), L (*CD*4+ *T* cells), *C*, *T*−*reg* cells (chemotherapy PAC-1), and OV-virus. These features were appended to the clinical data for subsequent ML analysis.

ML algorithms were applied to each MRI time point using current and previous data. Linear regression was first conducted with the merged dataset via *fitlm* in *Matlab*, generating RMSE and p-values to assess feature significance. The data were then discretized into tertiles for neural network training with 50 neurons and repeated 100 times to calculate the average performance from confusion matrices.

### 3.2 Datasets with tumor size as a categorical variable

This approach was extended to two datasets reporting tumor size categorically. The third dataset included 626 young women with ovarian cancer, providing data on age, nulliparity, contraceptive use, menopause, family history, full-term pregnancies, obesity, metastasis, *lymph* node status, *PIK*3*CA* and *TP*53 mutations, tumor size, lymph nodes, histology, vascular invasion, grade, adjuvant chemotherapy, radiotherapy, hormone therapy, and progression.

The fourth dataset comprised 41, 000 ovarian cancer cases with extensive clinical and treatment information, including metastasis, age, *lymph* node status, *PIK*3*CA*, *P*53, *BRCA*, stage, nodal status, histology, tumor size, grade, surgical margins, surgeries, chemotherapy, antihormonal, and other treatments. Tumor size was coded as categories 1, 2 or 3.

Following data pruning for chemotherapy regimen consistency, these datasets contained 41,000 and 626 samples. Each tertile group was assigned a random number between 0 and 100 as an initial tumor size condition, and the model was numerically solved for each sample, as described for continuous data.

Solution vectors at each time point for variables such as *D*, *T* − *reg* cells, *C*, *BRCA*, and chemotherapy drugs, were converted into categorical indices and merged with clinical data. As this was a classification problem, neural network algorithms were applied exclusively.

### 3.3 ML model

To optimize the treatment of granulosa cell tumors of the ovary using a TRAIL-producing oncolytic viruses and PAC-1 therapy through precise tumor size prediction, the *ML* model was trained using prior tumor size data:

For the 10,389-patient continuous dataset:

Predict *Volume* 2 (second MRI tumor size) from all data plus *Volume* 1 (first MRI),

Predict *Volume* 3 (third MRI) from all data plus *SER* *Volume* 1 (first MRI),

Predict *Volume* 3 from all data plus *Volume* 1 and *Volume* 2.

For categorical datasets, tumor size was predicted once per dataset based on clinical data and baseline measurements.

## 4 Results and discussion

**Figure 1:**
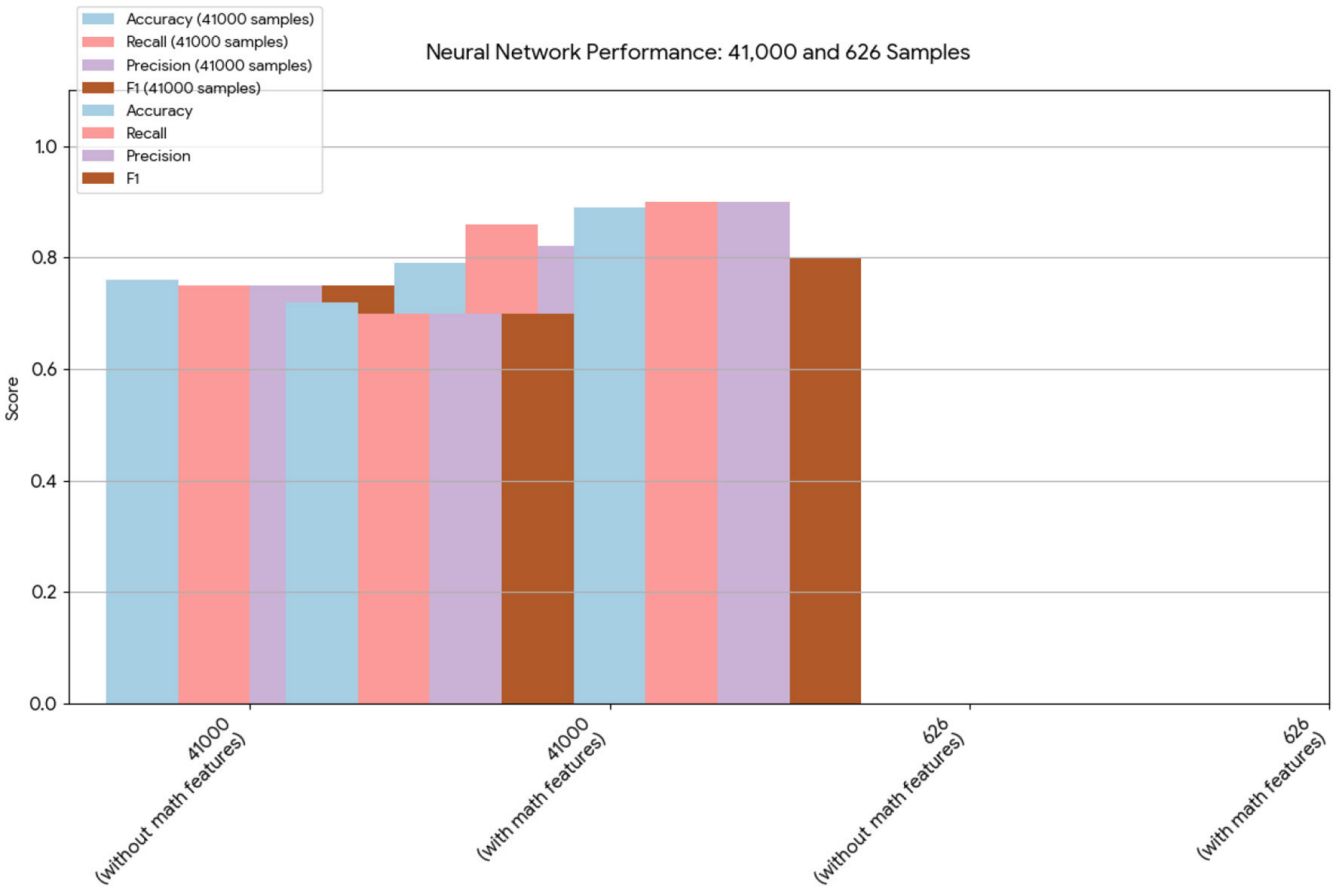
This chart provides a direct comparison of the neural network results on the 41,000- and 626-sample datasets. In both cases, the models that included the mathematical model features demonstrated improved overall performance, particularly in terms of accuracy and recall.

**Figure 2:**
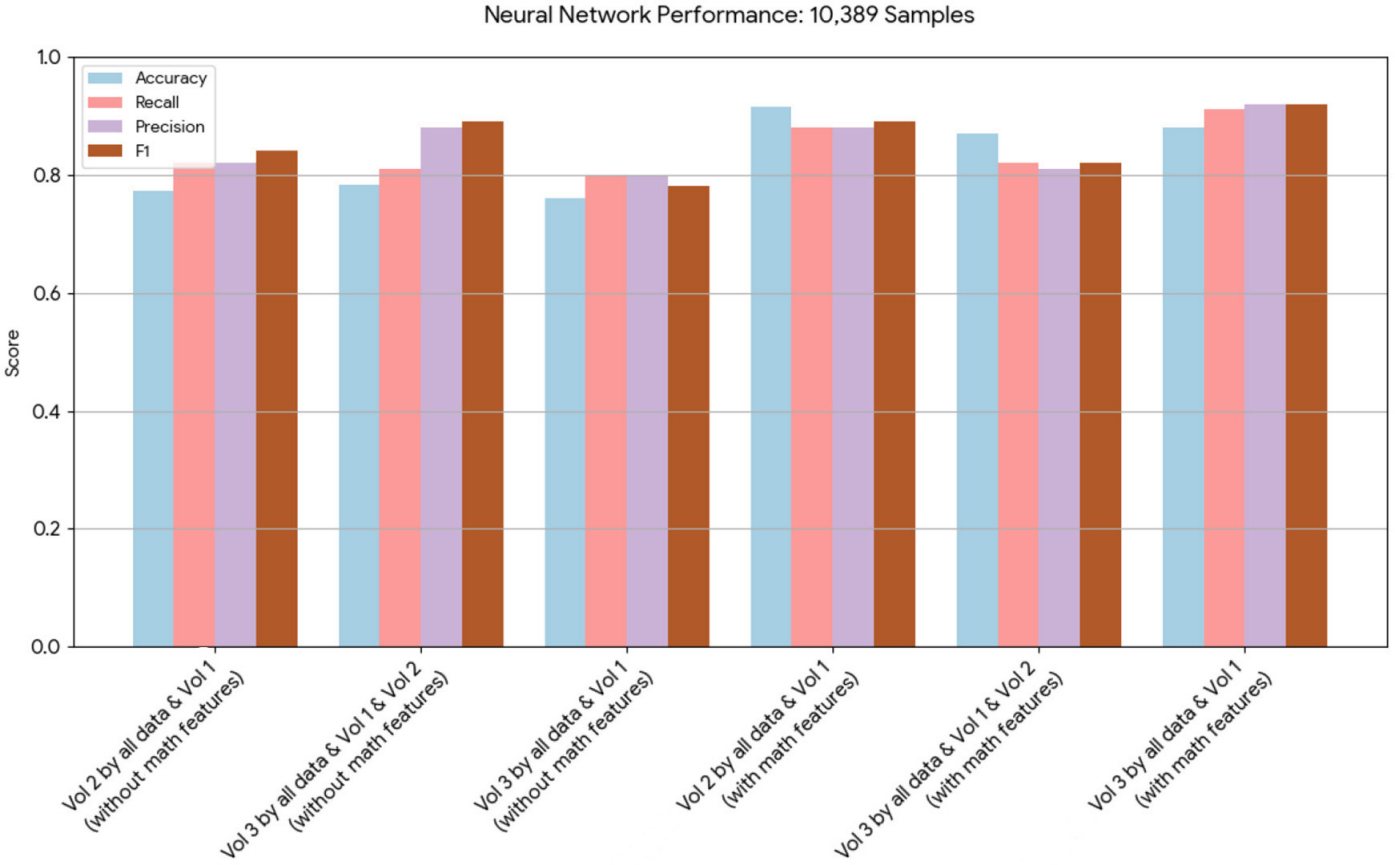
This chart displays the performance metrics of neural network models run on a dataset of 10,389 samples. The models with mathematical model features consistently show a notable increase in performance across all metrics compared to the models without.

**Figure 3:**
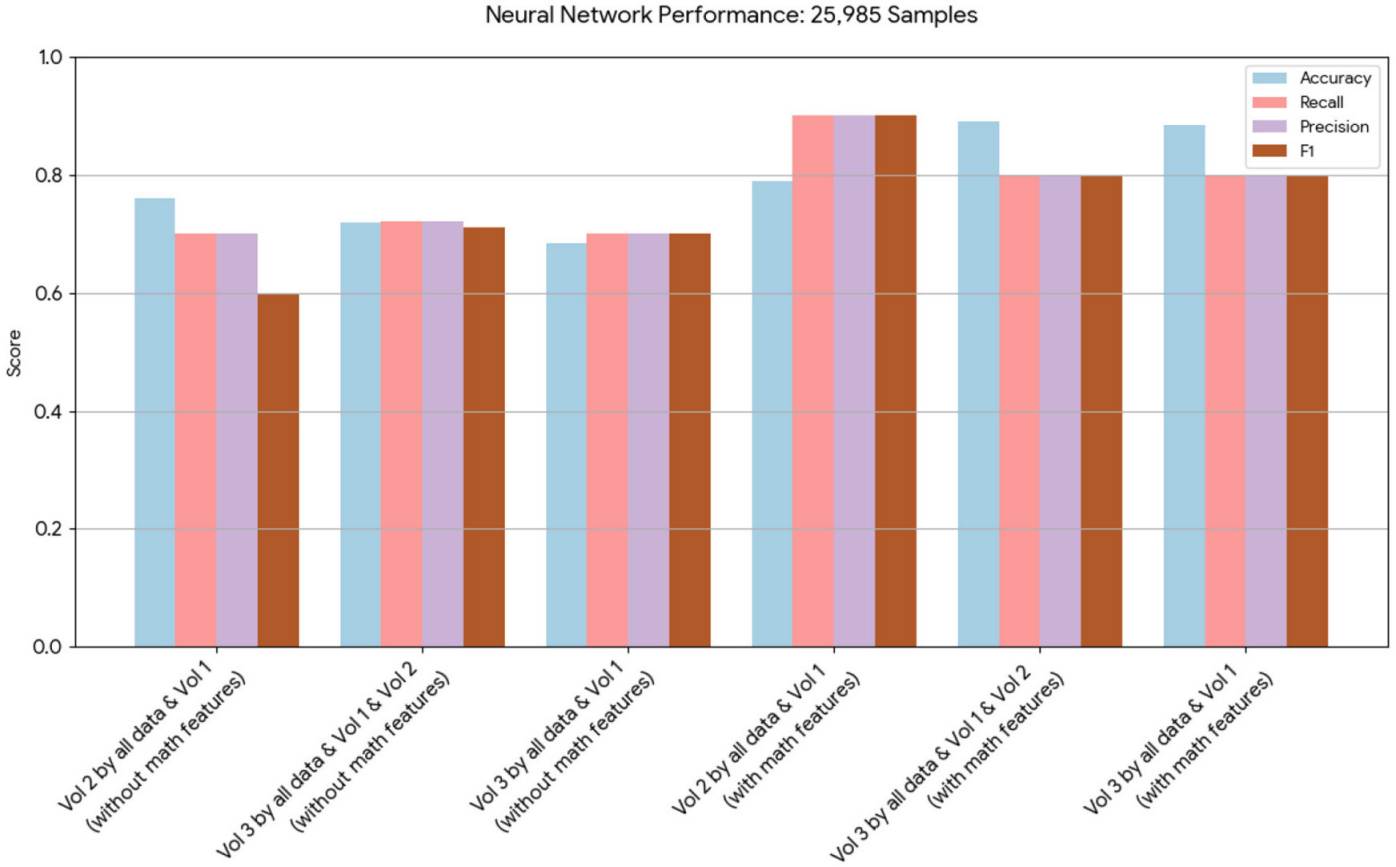
This chart presents the performance of neural network models on 25,985 samples. It highlights that, while both sets of models performed well, those incorporating the mathematical model features achieved higher scores in terms of accuracy, recall, and precision.

**Figure 4:**
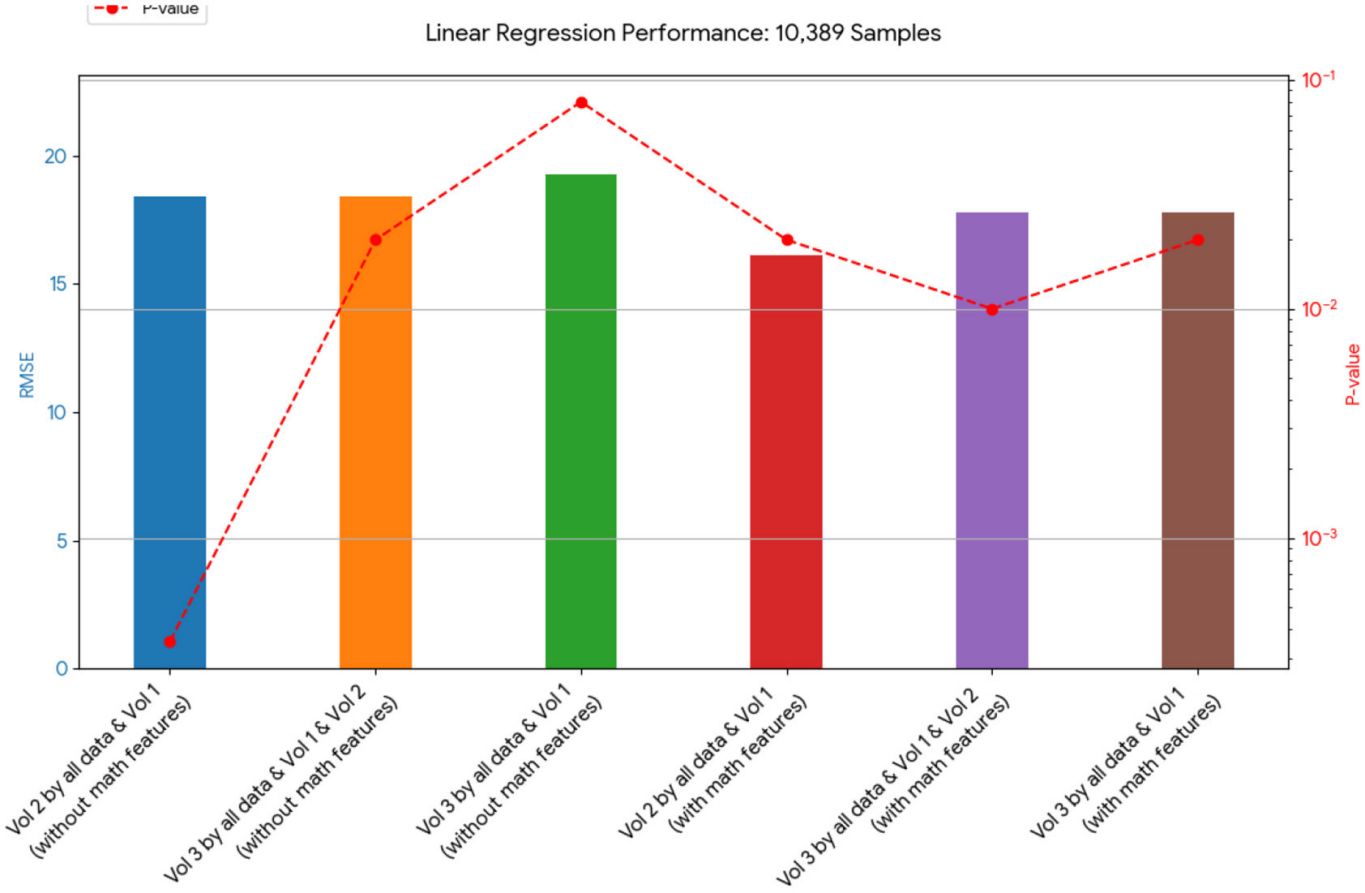
This chart shows the RMSE of linear regression models on the 10,389-sample dataset. The models that included the mathematical model generally had a lower RMSE, indicating a better fit to the data than those without it.

**Figure 5:**
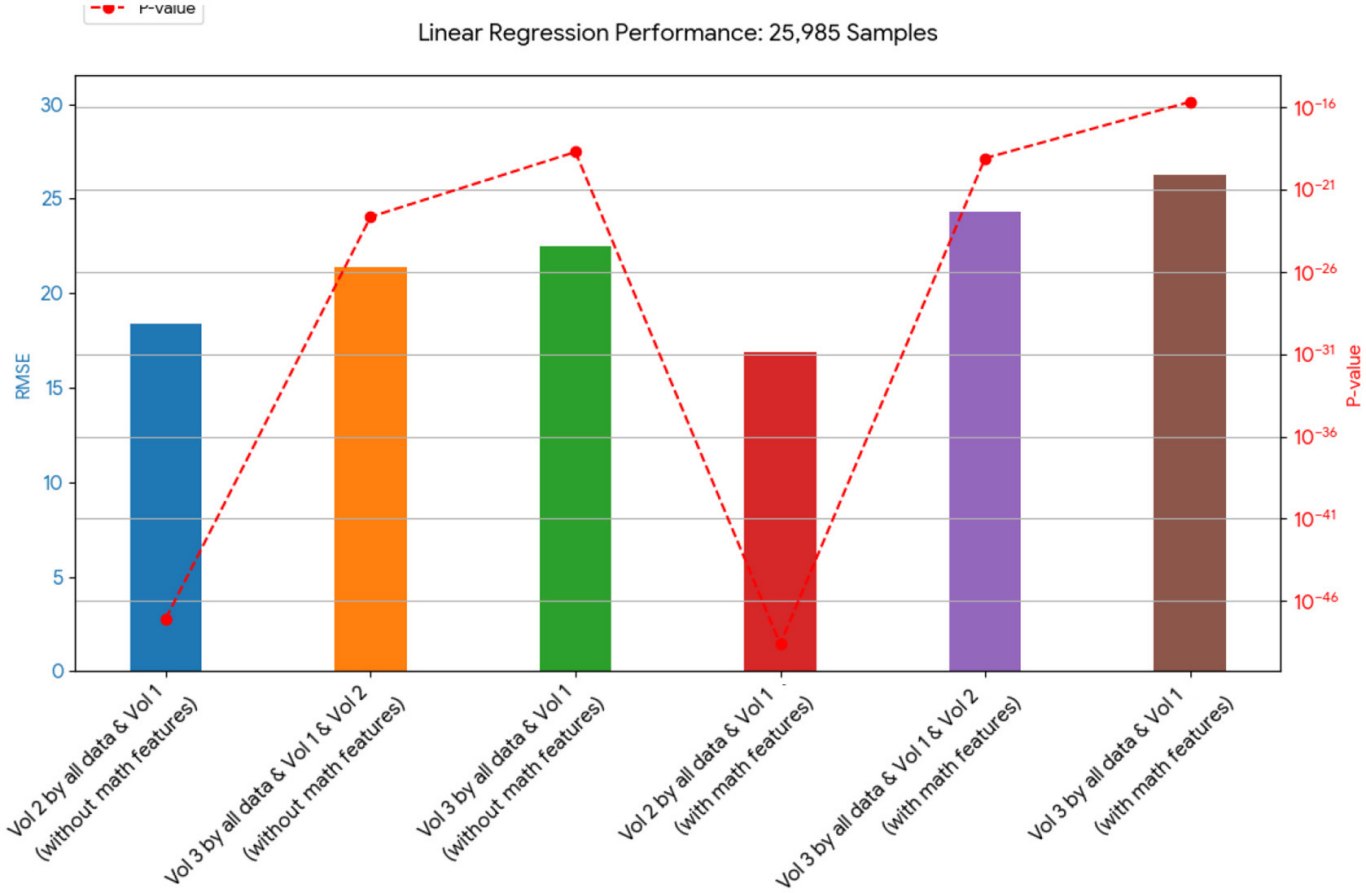
This chart presents the RMSE of linear regression models on 25,985 samples. It shows a varied outcome, with some models containing mathematical features performing better and some performing worse in terms of RMSE than their counterparts without these features.

A novel method integrating mathematical model outputs with clinical data was developed to improve tumor size prediction accuracy for granulosa cell tumor treatment with TRAIL-producing oncolytic virus and PAC-1 therapy. Linear regression and neural networks were applied to four ovarian cancer datasets, each offering unique advantages. Linear regression provided direct size predictions, while neural networks classified tumors into defined ranges. The results are presented in Tables 4–19. In Figures 1–5 we summarize the model and experimental data as histograms: Figures 1–3 define the model's structure and drug characteristics (neural network performance), while Figures 4, 5 present linear regression results for tumor size prediction, comparing models without and with mathematical features, respectively, to demonstrate improved accuracy.

**Table 4:** Linear regression results using machine learning.

**Volume 3 by all data and Volume 1**		
**Performance**	**RMSE**	***P*** **-value**
	19.3	0.08
**Significant features**		
	Θ**-Values**	***P*** **-values**
Volume1	0.4	0.0087

Without the results from the mathematical model.

**Table 5:** Linear regression results using machine learning.

**Volume 2 by all data and Volume 1**		
**Performance**	**RMSE**	***P*** **-value**
	16.1	0.02
**Significant features**		
	Θ**-Values**	***P*** **-values**
Volume 1	0.501	0.001
**Volume 3 by all data and Volume 1 and Volume 2**		
**Performance**	**RMSE**	***P*** **-value**
	17.8	0.01
	Θ**-Values**	***P*** **-values**
BRCA	−52.85	0.008
Lymph_Node positive	−22.234	0.004
PIK3 mutation	17.77	0.02
Volume2	0.43	0.01
M101	6.63	0.001
**Volume 3 by all data and Volume 1**		
**Performance**	**RMSE**	***P*** **-value**
	17.8	0.02
**Significant features**		
	Θ**-values**	***P*** **-values**
Lymph_Node positive	−18.631	0.01
PIK3 mutation	14.39	0.06
Volume1	0.4	0.01
M101	5.82	0.01

With the results from the mathematical model.

**Table 6:** Neural networks results using machine learning.

	**Volume 2 by all data and Volume 1**
Accuracy	77.3%
Recall	0.82
Precision	0.82
F1	0.84

Without the results from the mathematical model. Results from 10,389 women with ovarian cancer.

**Table 7:** Neural networks results using machine learning.

	**Volume 3 by all data and Volume 1 and Volume 2**
Accuracy	78.34%
Recall	0.81
Precision	0.88
F1	0.89

Without the results from the mathematical model. Results from 10,389 women with ovarian cancer.

**Table 8:** Neural networks results using machine learning.

	**Volume 3 by all data and Volume 1**
Accuracy	76%
Recall	0.8
Precision	0.8
F1	0.78

Without the results from the mathematical model.

**Table 9:** Neural networks results using machine learning.

	**Volume 2 by all data and Volume 1**
Accuracy	91.42%
Recall	0.88
Precision	0.88
F1	0.89

With the results from the mathematical model. Results from 10,389 women with ovarian cancer.

**Table 10:** Neural networks results using machine learning.

	**Volume 3 by all data and Volume 1 and Volume 2**
Accuracy	87%
Recall	0.82
Precision	0.81
F1	0.82

With the results from the mathematical model. Results from 10,389 women with ovarian cancer.

**Table 11:** Neural networks results using machine learning.

	**Volume 3 by all data and Volume 1**
Accuracy	87.93%
Recall	0.91
Precision	0.92
F1	0.92

With the results from the mathematical model.

**Table 12:** Linear regression results using machine learning.

**Volume 2 by all data and Volume 1**		
**Performance**	**RMSE**	***P*** **-value**
	18.4	8.24·10^−48^
**Significant features**		
	Θ**-Values**	***P*** **-values**
TP53 mutation	20.88	0.04
MRI	0.769	2.32·10^−49^
**Volume 3 by all data and Volume 1 and Volume 2**		
**Performance**	**RMSE**	***P*** **-value**
	21.4	2.23·10^−23^
**Significant features**		
	Θ**-Values**	***P*** **-values**
BMI	−23.72	0.02
MRI	0.4	0.0004
MRI 2	0.4	0.002
**Volume 3 by all data and Volume 1**		
**Performance**	**RMSE**	***P*** **-value**
	22.5	1.96·10^−19^
**Significant features**		
	Θ**-Values**	***P*** **-values**
BMI	−18.94	0.04
MRI	0.6	2.43·10^−22^

Without the results from the mathematical model.

**Table 13:** Linear regression results using machine learning.

**Volume 2 by all data and Volume 1**		
**Performance**	**RMSE**	***P*** **-value**
	16.9	2.34·10^−49^
**Significant features**		
	Θ**-Values**	***P*** **-values**
MRI	0.89	2.55·10^−58^
CD4+T	1.67·10^−9^	0.04
T-reg	−7.87·10^−9^	0.04
Dentritic cells	−0.29	0.03
**Volume 3 by all data and Volume 1 and Volume 2**		
**Performance**	**RMSE**	***P*** **-value**
	24.3	8.2·10^−20^
**Significant features**		
	Θ**-Values**	***P*** **-values**
BMI	−38.9	0.009
MRI	0.46	0.01
MRI2	0.48	0.0002
**Volume 3 by all data and Volume 1**		
**Performance**	**RMSE**	***P*** **-value**
	26.3	2.172·10^−16^
**Significant features**		
	Θ**-Values**	***P*** **-values**
BMI	−26.37	0.02
MRI	0.44	2.3·10^−18^

With the results from the mathematical model.

**Table 14:** Neural network results using machine learning.

	**Volume 2 by all data and Volume 1**
Accuracy	76.1%
Recall	0.7
Precision	0.7
F1	0.6

Without the results from the mathematical model. Results from 25,985 women with ovarian cancer.

**Table 15:** Neural network results using machine learning.

	**Volume 3 by all data and Volume 1 and Volume 2**
Accuracy	71.92%
Recall	0.72
Precision	0.72
F1	0.71

Without the results from the mathematical model. Results from 25,985 women with ovarian cancer.

**Table 16:** Neural network results using machine learning.

	**Volume 3 by all data and Volume 1**
Accuracy	68.4%
Recall	0.7
Precision	0.7
F1	0.7

Without the results from the mathematical model.

**Table 17:** Neural network results using machine learning.

	**Volume 2 by all data and Volume 1**
Accuracy	79%
Recall	0.9
Precision	0.9
F1	0.9

With the results from the mathematical model.

**Table 18:** Neural network results using machine learning.

	**Volume 3 by all data and Volume 1 and Volume 2**
Accuracy	89%
Recall	0.8
Precision	0.8
F1	0.8

With the results from the mathematical model.

**Table 19:** Neural network results using machine learning.

	**Volume 3 by all data and Volume 1**
Accuracy	88.39%
Recall	0.8
Precision	0.8
F1	0.8

With the results from the mathematical model.

### 4.1 Linear regression algorithm

Tables 4, 20, and 21 present the linear regression predictions without mathematical features (first dataset, 10,389 samples), whereas Table 5 includes these features. Although the p-values remained similar, the RMSE values decreased with the addition of mathematical features. For example, the RMSE for Volume 2 dropped from 18.4 (Tables 4, 20, 21) to 16.1 (Table 5), and for Volume 3, it decreased from 19.3 to 17.8.

**Table 20:** Linear regression results using machine learning.

**Volume 2 by all data and Volume 1**		
**Performance**	**RMSE**	***P*** **-value**
	18.4	0.000353
**Significant features**		
	Θ**-Values**	***P*** **-values**
Volume1	0.8896	1.23·10^−9^

Without the results from the mathematical model.

**Table 21:** Linear regression results using machine learning.

**Volume 3 by all data and Volume 1 and Volume 2**		
**Performance**	**RMSE**	***P*** **-value**
	18.4	0.02
**Significant features**		
	Θ**-Values**	***P*** **-values**
BRCA	−23.855	0.05
Volume2	0.5	0.02

Without the results from the mathematical model.

This indicates that incorporating mathematical model outputs into ML models improves prediction performance. Tables 4, 20, and 21 identified *BRCA* and *Volume* 2 as significant, while Table 5 additionally highlights *Lymph* and *PIK*3*CA*, providing further insights into treatment-relevant factors. Notably, the feature *M*101 (chemotherapy administered on day 101) was found to be significant, suggesting its potential role in personalized treatment adjustment.

Tables 12, 13 for the second dataset (25, 985 patients) showed improved p-values and identified *CD*4+ and *D* as important features when including mathematical outputs.

### 4.2 Neural network algorithm

Neural network performance metrics (*accuracy*, *recall*, *precision*, *F*1*score*) without mathematical features are reported in Tables 6–8, 14–16, 22, and 23. The results obtained with mathematical features are presented in Tables 9–11, 17–19, 24, and 25. For example, *Volume* 2 accuracy increased from 77.3% (*F*1*score* 0.84, Table 6) to 91.42% (*F*1*score* 0.89) when including mathematical model outputs.

**Table 22:** Data without the mathematical model features.

	**Size**
Accuracy	76.1%
Recall	0.75
Precision	0.75
F1	0.75

**Table 23:** Data without the mathematical model features.

	**size**
Accuracy	71.91%
Recall	0.7
Precision	0.7
F1	0.7

**Table 24:** Data with the mathematical model features.

	**The 101th vector**
Accuracy	79%
Recall	0.86
Precision	0.82
F1	0.84

**Table 25:** Data with the mathematical model features.

	**The 101th vector**
Accuracy	89%
Recall	0.9
Precision	0.9
F1	0.8

Similarly, the 25, 985-patient dataset showed improved results when mathematical features were integrated (Tables 17–19 vs. 14–16). For the third cohort (41,000 women), accuracy rose from 76.1% to 79%, with corresponding improvements in *recall*, *precision*, and *F*_1_.

Similarly, the 25, 985-patient dataset showed improved results when mathematical features were integrated (Tables 17–19 vs. 14–16). For the third cohort (41,000 women), accuracy rose from 76.1% to 79%, with corresponding improvements in *recall*, *precision*, and *F*_1_.

Overall, across all algorithms and datasets, merged data outperformed original clinical data alone. These findings highlight the value of incorporating mathematical model-derived features for more accurate prediction of tumor dynamics, supporting the effective and personalized treatment of granulosa cell tumors of the ovary using a TRAIL-producing oncolytic virus and PAC-1 therapy.

### 4.3 Sensitivity analysis

In this section, we evaluated how changes in specific model parameters could influence predicted outcomes. To do this, we conducted a local sensitivity analysis, systematically varying each parameter from −85% to 85% of its value.

We assessed the changes in the predicted final tumor volume relative to baseline simulations that used a consistent 21-day treatment course consisting of daily PAC-1 administration at 375 mg with an initial multiplicity of infection (MOI) of 0.03 applied to a tumor population of 10^9^ cells.

**Figure 6:**
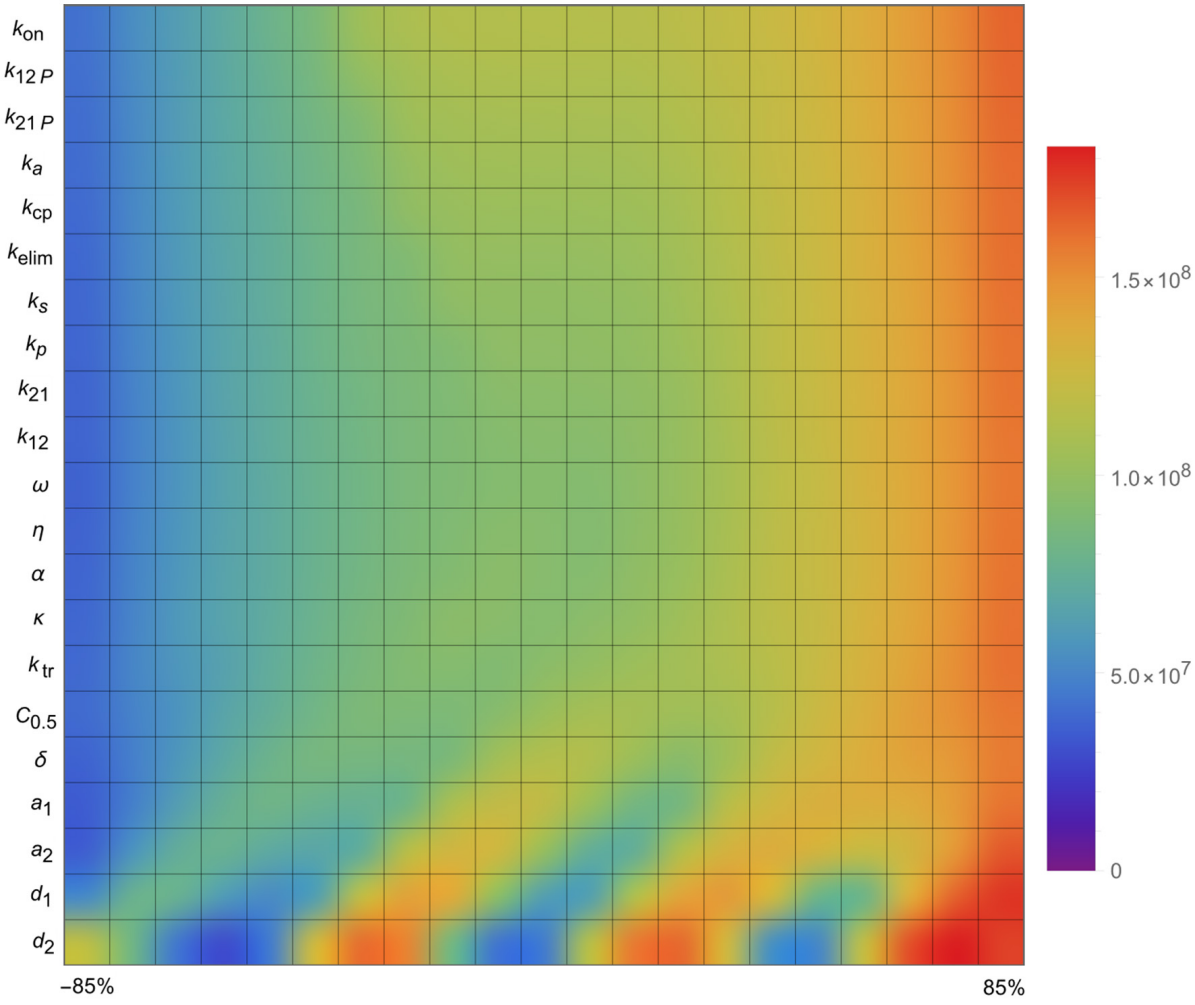
Sensitivity analysis of the parameters of the mathematical model, i.e., the impact of the parameters on tumor size.

The results of the sensitivity analysis, presented in Figure 6, revealed that only a limited number of parameters significantly impacted tumor progression in the model: *a*_1_, *a*_2_, *d*_1_, and *d*_2_. Specifically, the tumor proliferation rate *a*_1_ and the tumor cell death rate *d*_2_ were the most influential, directly affecting tumor expansion.

Quite surprisingly, the other parameters (in the list presented in Figure 6) did not affect the stability of the model in general and the size of the tumor in particular, i.e., they appeared to have little effect overall.

These findings indicate that, beyond the initial tumor burden, the tumor's intrinsic growth characteristics-particularly its proliferation rate-are critical indicators of how well a combination treatment might perform.

## 5 Conclusions

In this study, we presented an innovative artificial analysis framework that integrates a mechanistic mathematical model with machine learning (ML) algorithms to improve prediction of tumor dynamics in the treatment of granulosa cell tumors of the ovary using the combined action of a TRAIL-producing oncolytic virus and PAC-1 therapy. By leveraging four extensive datasets containing both continuous and categorical tumor size data, our approach systematically combined personalized mathematical simulations with clinical and imaging features to enhance ML predictive performance.

Our results demonstrated that incorporating features derived from the mathematical model consistently improved prediction accuracy across all datasets and ML approaches used. Linear regression models showed a marked reduction in root mean square error (RMSE) when mathematical outputs were added, while neural network models exhibited increased accuracy, precision, recall, and F1 scores. These improvements underscore the importance of integrating mechanistic insights with data-driven algorithms for reliable tumor burden prediction.

The inclusion of mechanistic variables such as immune cell dynamics, the pharmacokinetics of PAC-1 and TRAIL, and tumor-virus interactions provided additional biologically relevant features that pure clinical data alone could not offer. This approach enables a more comprehensive representation of tumor behavior under therapy, enhancing the potential for effective personalized treatment strategies in granulosa cell tumors of the ovary.

However, several limitations should be acknowledged. First, while the mathematical model included key tumor-immune-pharmacokinetic interactions, further refinement and validation with larger prospective clinical datasets are needed to generalize these findings. Second, although this framework focused on granulosa cell tumors treated with a TRAIL-producing oncolytic virus and PAC-1, extending the methodology to other tumor types and therapeutic combinations could broaden its clinical applicability.

This study introduces an innovative hybrid framework that integrates mechanistic mathematical modeling with machine learning (ML) to predict tumor dynamics in granulosa cell tumors treated with a TRAIL-producing oncolytic virus and PAC-1 therapy. Unlike conventional approaches that rely solely on clinical and imaging data, this method enriches ML models with biologically meaningful variables derived from tumor–immune–drug interaction simulations. This integration significantly improves prediction accuracy, precision, recall, and F1 scores across multiple large datasets. Our work not only demonstrates the added value of combining mathematical and data-driven approaches but also establishes a novel proof-of-concept for personalized, mechanism-informed treatment planning in rare ovarian cancers where therapeutic options are limited.

In conclusion, the proposed artificial analysis framework represents a promising tool for precision oncology. By combining mathematical modeling and ML algorithms, clinicians and researchers can gain deeper insights into tumor dynamics, optimize treatment planning, and potentially improve outcomes for patients with granulosa cell tumors of the ovary. Future studies should focus on integrating this framework into clinical decision-support systems and exploring its use in real-time treatment adaptation.

## Data Availability

The original contributions presented in the study are included in the article/supplementary material; further inquiries can be directed to the corresponding author.
